# Cultural big data: nineteenth to twenty-first century panoramic visualization

**DOI:** 10.3389/fdata.2024.1309887

**Published:** 2024-11-08

**Authors:** Tsz Kin Chau, Paul Bourke, Lily Hibberd, Daniel Jaquet, Sarah Kenderdine

**Affiliations:** ^1^Laboratory for Experimental Museology, College of Humanities, Digital Humanities Institute, Swiss Federal Polytechnical Institute of Technology (EPFL), Lausanne, Switzerland; ^2^Private Consultant, Melbourne, VIC, Australia

**Keywords:** big data, panorama, battle of Murten, gigapixel image, data visualization, 3D augmentation, media archaeology, cultural history

## Abstract

From the nineteenth-century panorama to the emergence of the digital panoramic format in the 1990's, the visualization of large images frequently relies on panoramic viewing strategies. Originally rendered in the form of epic painted canvases, these strategies are now amplified through gigapixel imaging, computer vision and machine learning. Whether for scientific analysis, dissemination, or to visualize cultural big data, panoramic strategies pivot on the illusion of immersion. The latter is achieved through human-centered design situated within a large-scale environment combined with a multi-sensory experience spanning sight, sound, touch, and smell. In this article, we present the original research undertaken to realize a digital twin of the 1894 panorama of the battle of Murten. Following a brief history of the panorama, the methods and technological framework systems developed for Murten panorama's visualization are delineated. Novel visualization methodologies are further discussed, including how to create the illusion of immersion for the world's largest image of a single physical object and its cultural big data. We also present the visualization strategies developed for the augmentation of the layered narratives and histories embedded in the final interactive viewing experience of the Murten panorama. This article offers researchers in heritage big data new schemas for the visualization and augmentation of gigapixel images in digital panoramas.

## 1 Introduction

Panoramic viewing strategies have been an integral part of massive image visualization from the nineteenth-century panorama to the emergence of the digital panorama in the 1990's. These strategies were first expressed as large-scale painted canvases, but gigapixel imagery, computer vision, and machine learning have since enhanced panoramic imaging techniques. Panoramic strategies can be employed in the study of scientific datasets or for the visualization and dissemination of cultural big data, all of which depend on creating the illusion of immersion in a computer-generated environment. The illusion of immersion is a consistently essential requirement across all platforms and uses, equally for scientific analysis, dissemination and entertainment, or as a form of access to cultural big data. The success of this illusion pivots on a large-scale visualization platform grounded in human-centered design and a multisensory experience that can encompass sight, hearing, touch or smell.

In parallel, cultural big data visualization is revolutionizing access to vast volumes of digitized cultural archives, enabling novel forms of presentation, interpretation and understanding. The data accumulated in these archives derive from various formats and sources that may or may not have an apparent collection theme. The Europeana project has for example aggregated around 60 million digitized heritage items in various media and formats, with collection themes ranging from “Industrial Heritage” and “Migration” to broader categories of “Art” and “Fashion” (Europeana, (n.d.)). These archives often lack coherent metadata curation, necessitating significant work to augment and transform the data points into a linkable dataset, as discussed in Alliata et al. (2023). This process is comparable to the data lake analogy in big data repositories (Sundaram and Vidhya, 2016). Given the immense and heterogeneous nature of such archives, traditional retrieval systems cannot optimally access the full content of their collections, revealing only a superficial fraction of the complete collection.

The GLAM sector, comprising galleries, libraries, archives, and museums, has recently embraced the use of the digital panorama and its derivatives as a crucial apparatus for safeguarding or promoting cultural heritage sites, performances or objects. Shifting museological and curatorial values have also prompted the GLAM sector to open their archives to the public and to adopt new perspectives such as participatory, responsive, reflexive, inclusive, interrogative, relational, and activist engagement with their publics (Kenderdine, 2021). This transformation places the audience in an active role, creating a demand to reimagine a generous browsing interface (Whitelaw, 2015) that caters to the critical and curious minds of the information flaneur (Dörk et al., 2011). As Whitelaw suggests, a humanistic interface to an archival collection should “reveal the scale and complexity of digital heritage collections” and “emphasize exploration and interpretation over task and information retrieval” (Whitelaw, 2015, p. 1). While direct and immersive interaction with large volumes of digitized images or with very large images, the panoramic format is self-evident, for text-based data it is less apparently advantageous. Moreover, it is important to note that some cultural archives are still under copyright and can only be made accessible within the institutional GLAM building or as part of special exhibitions that protect the data from copyright infringement. Computational methods can assist in this context, to generate new ways for audiences to access the depths of a vast dataset through situated museological experiences for cultural big data visualization, thereby enriching engagement with digitized collections.

Cultural big data visualization further provides scalable interfaces for large data archives in their entirety, which foster access to generous volumes of data rather than a portion or sample. The 2-fold computational avenues of interactive immersion and data science are fundamental to these interfaces. Several multimodal visualization projects and specialized systems demonstrate the evolution of the state-of-the-art in this domain. *T_Visionarium II* (2006) is an interactive immersive installation operating within the Advanced Interaction and Visualization Environment (AVIE), an omnidirectional stereoscopic interactive environment. In this project, 24 h of television footage have been transformed into a database of 22,500 video clips annotated with keywords. Over 200 videos play simultaneously on the omnidirectional screen. When a user selects an item, the videos on the screen rearrange according to the semantic similarity of the selected clip, following the parametric weighting of color descriptors and tags (McGinity et al., 2007). Another more recent project, *Jazz Luminaries* (2019), features 13,000 videos from the Montreux Jazz Archive displayed in a full dome structure. Data science techniques has contributed to this project to generate a network diagram of 5,400 musicians who have played at the Montreux Jazz Festival, with each node representing an artist and each link a collaboration. Participants can explore the network with a spherical controller that allows them to activate the visualization by scrolling over a specific artist's node that plays a corresponding music track (Kenderdine, 2021). In both these examples, participants can easily glimpse the latent structure of a vast dataset in conjunction with a serendipitous exploration that allows for the personalized, unique remix of the dataset.

Beyond enhancing access to archival collections, cultural big data visualization has various applications for research and curatorial support. One notable example is *Blue Dots 360* (2012), an omnidirectional data browser developed with the University of California Berkeley as an extension to the *Blue Dots* project that also runs on the AVIE. This project encompasses a massive searchable digitized corpus of the Koryo version of the Tripitaka Koreana, a Chinese Buddhist Canon. In this visualization, each glyph is represented by a blue dot that changes color when a search term is matched. The *Blue Dots 360* system fills the 3D space of the omnidirectional screen with the corpus, visualizing search patterns and enabling researchers to identify new linguistic patterns and relationships within the text (Kenderdine et al., 2013). Another significant example of cultural big data visualization is *mArchive* (2014), an omnidirectional installation featuring 100,000 image records from Museum Victoria (Sarah Kenderdine). The *mArchive* application connects objects through collection metadata which is designed as a real-time curating machine that allows participants to discover emergent narratives and enhance their ability to explore and interpret large digitized museum collections (Kenderdine et al., 2021).

This article presents original research into cultural big data visualization undertaken to realize a digital twin of the 1894 panorama of the battle of Murten as part of the *Digitizing and Augmenting the Panorama of the Battle of Murten* (DIAGRAM) project, led by EPFL Laboratory of Experimental Museology (eM+) with the Foundation for the Panorama of the Battle of Murten, which commenced in 2022. Our research for this project demonstrates how the world's largest image of a single physical object is generating unique forms of visualization for cultural big data. It describes the affordances for viewers experiencing an ultra-high-definition digitized nineteenth-century panorama, as well as new approaches to qualitative and quantitative data analysis and exploration for *in-situ* exploration of its big data. The DIAGRAM project also illuminates how multisensory experiences in large-scale panoramic environments depend on human-centered design to evoke senses of sight, sound, touch, and smell. The resulting installation of the Murten visualization will be exhibited in 2025 and 2026 in several Swiss museums to commemorate the 550th anniversary of the Burgundian Wars, of which the battle of Murten (1476) was the main event.^1^

Before introducing the DIAGRAM project, we begin with a short history of panoramic viewing strategies inaugurated during the golden era of painted panoramas in the late-nineteenth century, followed by the emergence of the panoramic format in photography up to the use of gigapixel images—approaches that underpin the visualization of the Murten panorama. Following this overview of the panorama's history, we outline the techniques and technological framework created for the visualization of the Murten panorama. Other innovative visualization techniques of the DIAGRAM project are addressed, such as how to produce an illusion of immersion for the largest image of a single physical object in the world and its cultural big data. We also demonstrate the visualization techniques devised to enhance the multi-layered stories and histories featured in the Murten panorama's final interactive viewing experience. These combined techniques and methods provide new schemas for the visualization and augmentation of gigapixel images in digital panoramas for researchers in heritage big data.

## 2 Historical panoramic viewing strategies and technological frameworks

Omnidirectional or panoramic viewing strategies have been pivotal to the visualization of very large images, from grand painted canvases to gigapixel images and have been used as paradigms for visualizing cultural big data. These strategies can be traced back to the nineteenth-century panorama through to the panoramic digital image format of the 1990's, which is now being augmented through computer vision and machine learning.

Following the 1790 rotunda at London's Leicester Square, painted panoramas peaked in popularity between 1830 and 1900, before declining with the advent of cinema in the early twentieth century (Panorama Council). In its prime, the panorama was a highly popular proto-cinematic attraction, which constituted for Oettermann (1997, p. 7) “first true mass medium.” The format originates from Robert Barker's patented design in 1787, which was designed to immerse viewers in the center of a scene, surrounded by a large-scale painted canvas in a purpose-built circular building.^2^ Various iterations of the panoramic concept were given different names throughout the nineteenth to beginning of the twentieth century. Most of them ended with the suffix -orama, derived from the Greek Öρ*αμα* (hórama), meaning “that which is seen, visible object, sight.” The prefix “pan” (Greek Πάν, pán) means “all,” so that “panorama” stands for “all which is to be seen,” or the “complete view” in simple English.

Three main subjects were pivotal to the panoramic paintings of the nineteenth century. The first was the landscape, or cityscape, which offered a romantic vision of a world where industrial development was taking on a disconcerting importance. The second was travel and the representation of distant, exotic lands, against a backdrop of colonialism (Comment, 2000; Oettermann, 1997). The third was the commemoration of military events, largely created for propaganda purposes and a world in conflict. This is the theme that the battle of Murten panorama represents, the case study for this article.

The digital techniques of the 1990's prompted a revival of panoramic immersion, initially in domains of media art but more recently in cultural heritage and entertainment sectors (Kenderdine, 2007). Despite these breaks in the history of panoramic seeing, we suggest that the early panorama phenomenon constitutes a predecessor for cultural big data visualization today. This, we contend, is due to the extent of the visual information painted on these early monumental canvases, which is a common contemporary viewing strategy, as examined in the following section.

### 2.1 The illusionistic trick: fooling the senses

Images have always been subject to technologies of spatial illusion, immersion, and display. As Grau (2002, p. 5) writes: “every epoch uses whatever means available to create maximum illusion.” Yet, the illusion of immersion is specifically achieved through the subjugation of a range of the spectator's senses to the total experience of the visualization, including sight, smell or hearing. The success of the illusion depends on creating a complete and coherent role for the viewer's body in the experience. In the theorization of immersive virtual environments, this completeness should evoke a sense of “presence” in which the mind allows the body to feel it is translocated within the fictional, represented space while remaining physically placed in the real world (McGinity, 2014).

Among the various experiences contributing to the culture of the spectacle that burgeoned in the first half of the nineteenth century, the panorama was the most impressive. Its illusion was based on the anamorphosis effect (a distorted projection), which was designed to produce an illusion in which the viewer felt as if they were in the center of the scene.^3^ Bending the frameless painting around 360 degrees caused the picture's surface and its perspectival plane to be reframed around the viewer, providing an unprecedented sense of depth, but also the potential for dizziness or nausea. The illusion was reinforced by the capacity of the user of the apparatus to isolate and control what it was possible to see.

The specifications of the purpose-built circular building in which the painting was hung included overhead natural lighting, ventilation without any windows, and a freestanding central viewing platform suspended around the vertical center point of the canvas, which spectators entered from underneath the structure. The platform also prevented the audience from approaching the picture. The edges of the image were covered by a canopy that concealed the sunlight as a source, maximizing the illusion of depth. In addition, the space at the bottom of the canvas under the viewing platform included three dimensional props (mannequins, cardboard cutouts) on top of a decor including natural or fabricated elements called the “faux-terrain” (see Oettermann, 1997, p. 130–144 and 187–221). Different types of live performance were sometimes staged on the faux-terrain or on the observation platform, such as music, dance, theatrical performances or olfactory events, as well as mechanical elements such as mobilizing the platform or modifying the light or atmosphere in the space.

Among the many innovations of the panorama is the involvement of the spectator's embodied presence in the enlivenment of the visualization. The patent for Robert Barker's panorama, for instance, included a technique for the image display to magnify the optical illusion by involving the visitor's body in the experience. Distinct from the panorama, the diorama was a hybrid form of painting and theater made of large flat or curved paintings to be viewed by an audience that was usually seated (Thomas, 2005). When a viewer and their body are active, and they free to interact with the content presented, they are drawn into greater cognitive, affective, and intellectual proximity with the illusion. In contrast, being seated, or alone in a parlor, tends to drastically reduce the immersion effect, not only because of diminished scales. Similarly, recent technologies that isolate the viewer from their environment, most evidently the virtual reality headset, separate the viewer from their body, and paradoxically weaken the embodied experience. Despite ever-greater image resolution, when it comes to creating illusion and ensuring a sense of immersion, the technologies used in the various mass media spectacles are of little value if the role of the visitor's body and senses are not taken into account in the design of the experience (on interaction design or a human/user-centered approach (see Reunanen et al., 2015).

The addition of sounds and smells to the repertoire of illusory tricks in panoramas are often overlooked as two powerful factors that can augment the sense of immersion (see Doornbusch, 2004). Sound was already implemented in early-nineteenth century panoramas, mainly with live performances by musicians, but were more often included in dioramas (Fehr, 2019). Smell was also implemented in some early systems such as the panorama and diorama, but it was rather exceptional and usually only featured during performances or live events such as in the Maréorama created by d'Alesi for the 1900 Paris World Exhibition (Deckers, 2021). According to Comment (2000), many audiences critiquing the panorama highlighted the lack of sound and even the absence of wind.

### 2.2 More than what meets the eye: augmenting panoramic scale, action, and data

The panoramic spectacles of the nineteenth and twentieth centuries pivoted on size and scale. These two factors also play a crucial role today in the analysis of the quantity and quality of the content presented to the public in installations of single or multiple works. For the panorama paintings, there was no standardized dimension other than their convincing ability to simultaneously immerse large groups of people. However, in the last quarter of the nineteenth century some companies began to operate panoramas across several locations, forming networks that required restricted dimensions. Since there were no global standards at the time, the dimensions ranged from 9 to 15 meters high and around 100 m long, and even longer for moving panoramas.^4^ This observation is relevant for installations with one (large) artwork, but there were many other spectacles presenting multiple works, such as the Cosmorama, which presented a large series of flat paintings viewed through a magnifying glass, presented in the Palais-Royal between 1808 and 1832, or the Great Exhibitions that toured panoramic works to multiple locations (Geppert, 2010).^5^

Early immersive media spectacles often featured accompanying documentation with additional information about the artwork or an experience (visitors' guides, program leaflets, etc.). Some were brief, to minimize the cost of production, others were ambitious, even book sized. Landscapes or cityscapes were accompanied by descriptions of places, while military scenes featured narration or witness accounts, especially if the battle was contemporaneous with the artwork. Both types of graphical or written documentation relied on the respective literary genre in fashion at the time, such as travel literature or the commemoration of battles (Sternberger and Neugroschel, 1977).

**Figure 1:**
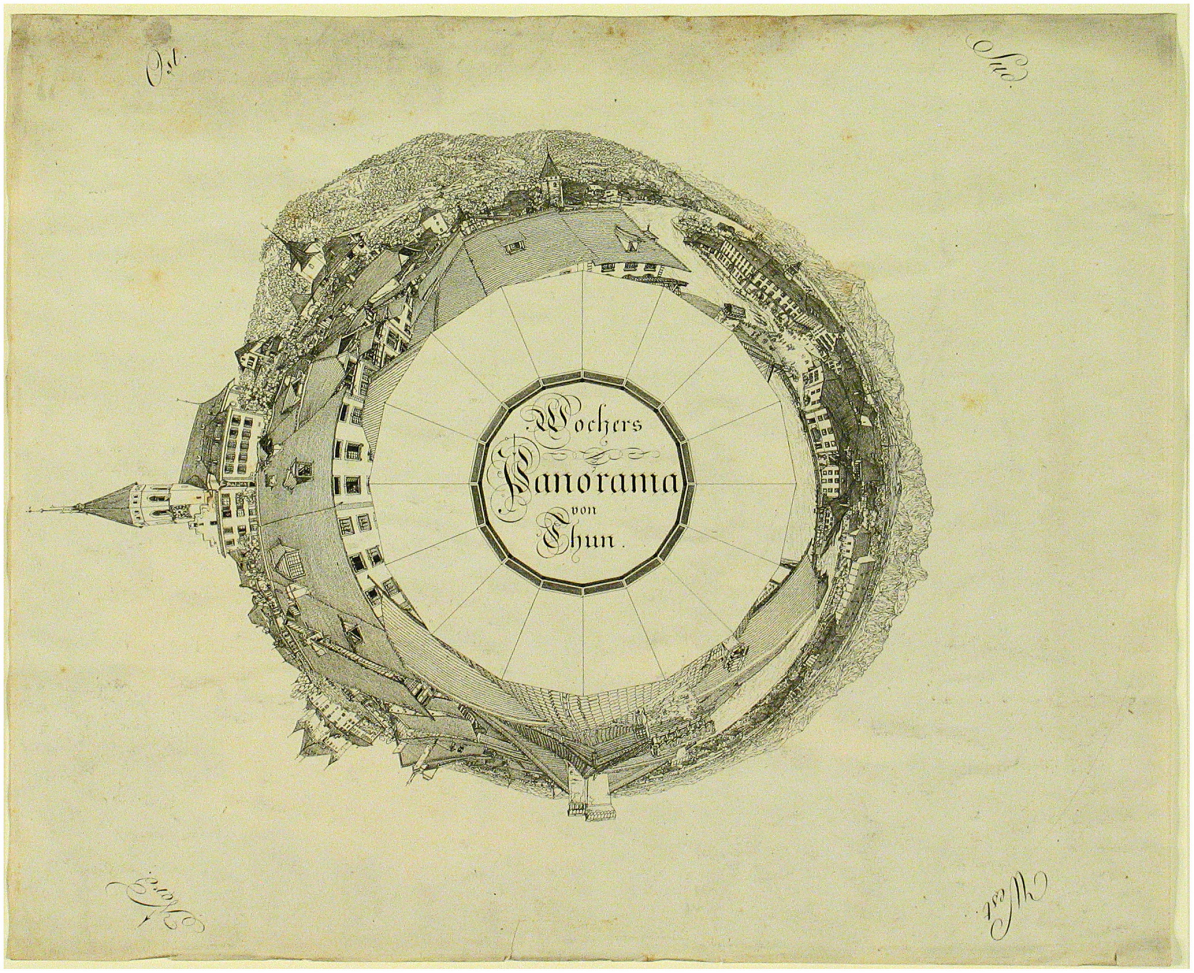
Orientation plan by Marquard Wocher for the Thun Panorama (1814). Image credits: Thun, Kunstmuseum.

Visual innovations were developed to include information on a 2D surface related to a surrounding panorama painting in a cylindrical form, allowing the visitor to locate the data relevant to a specific scene. The Wocher Panorama is the oldest preserved panorama in the world, dated 1814. It represents a cityscape of Thun and the surrounding alps (Ganz, 1975) and it is also notable for its visitor orientation plan (Figure 1), which includes a map with a legend shown in anamorphic optical deformation. This type of graphical guide was often used in landscape panoramas in the second half of the nineteenth century.

The most typical form of accompanying documentation for the panorama was however textual. The leaflet produced as a visitor's guide for the *Panorama of the battle of Murten* (1894) is a good example, though it was predominantly designed for light public entertainment, tinted with nationalistic discourse on the prowess of the Swiss warriors from the distant Middle Ages. It was in the octavo format, measured ~15 by 23 centimeters and contained six pages without any images.^6^ The text describes the battle scene with historical anecdotes, as well as the names of the main characters and places. It also includes a legend with numbered scenes in the panorama.

These examples demonstrate the limitations of interaction design and data visualization in the early panoramas because of the printed format. While the size and scale of the nineteenth-century panorama artworks and their accompanying documentation deserve to be called “big data,” the term takes on its full meaning with the transition to digital technologies and digital panoramas, which are the focus of the remainder of this article. Today, digital cultural heritage approaches are overcoming such limitations to provide meaningful avenues of access to data on various supports. Yet, the principles remain the same: big data in the domain of digital cultural heritage not only augments the optical or auditory experience of tangible and intangible to document significant sites, historical monuments, or artifacts, large amounts of data can also provide insights into art and aesthetics through processes of information translation and analysis. Furthermore, the visualization and analysis of cultural big data is harnessing sophisticated tools and techniques, such as computer vision and machine learning, to automatically process and analyze large numbers of images or the big data of very large digital files such as gigapixel images.

## 3 From the painted canvas to gigapixel images

### 3.1 The dawn of digital panoramas and their derivatives

With the birth of photography in the mid-nineteenth century, painted panoramas were revived in the form of panoramic vistas, realized by shooting a series of photos along a horizontal line and then manually stitching them together. The internet and advances in computer graphics in the 1990's and early 2000's set the stage for the next major evolution in digital panoramas. QuickTime VR (QTVR) software developed by Apple (1991) popularized panoramic or spherical maps (Chen, 1995; Kenderdine, 2007). Interactive panoramas, or 360-degree views, became a popular feature on websites, particularly for real estate, travel, and hospitality industries. Such panoramas offered viewers the ability to virtually look around a space, simulating the experience of being present in a place. The technology involved stitching together multiple photos, then mapping them onto a three-dimensional space that could be navigated using the computer mouse or keyboard.

The invention of the digital camera and associated image processing software in the late-twentieth century, marking a significant leap forward in the development of digital panoramas. The subsequent development of smartphones equipped with high-quality cameras and gyroscopes further propelled the progress of digital panoramas. Various mobile applications were developed to capture, stitch, and view panoramic photos directly on these devices, thereby democratizing the technology. Today, 360-degree video and virtual reality (VR) experiences are ubiquitous to digital panoramas. These systems not only provide a fully immersive visual experience, but they also incorporate elements such as sound, movement, and even touch, delivering a truly multisensory experience. Despite the rapid advancements in this field, the core principle remains the same as it was in the early painted panoramas or later photographic panoramas, as they enable viewers to appreciate the wider perspectives of the world around them.

The way that we see the world in relation to and in conjunction with art determines what we can distinguish, therefore what is knowable. Cohering fields of media theory and visual studies, Martin Jay in his seminal article “Scopic Regimes of Modernity” (Jay, 1988) identified three scopic regimes of ocular perception. The first two are based on Cartesian perspectivalism (dominant in Western culture) and the last is the art of describing a product of the Baroque era (Alpers, 1984). In digital panoramas and VR experiences, as in painted panoramas, all three scopic regimes can be woven together, while the illusion of immersion is achieved by tricking the eye, as outlined earlier. The orchestrated illusion of immersion and the presence of the viewer in the scene also possess both social and cultural dimensions (Champion, 2006). The broad use of digital panorama in the cultural heritage field has drawn particular attention to this issue, especially in relation to the core message of the visualization and the data made available to users, concerns that are pertinent to the Murten panorama (Kenderdine, 2007).

### 3.2 The emergence and uses of gigapixel images

Gigapixel images are digital images composed of billions of (10^9^) pixels.^7^ Their ultra-high resolution offers a level of detail that allows viewers to zoom into parts of the image without losing clarity, providing an immersive, interactive experience when optimally produced. Their compelling factor lies in the ability to discover minuscule details that would otherwise be invisible in standard resolution photos. The application of gigapixel images spans various fields and disciplines. For example, in art conservation, gigapixel imaging allows experts to forensically examine fine details of artwork, including brush strokes and color gradients, without physically touching the piece. Landscape architecture and astronomy are two other fields that have importantly contributed to the development of the gigapixel images.

Since the 1990's, there has been a remarkable evolution in technology and approaches for acquiring gigapixel images. The first example is *Great Wall West* (1999) by Jim Hellemn, which is claimed to be the first gigapixel image ever produced. This underwater photograph depicts a vertical coral reef wall, 6 m high by 21 m wide, in the Cayman Islands (Hellemn, (n.d.a),n). *Great Wall West* is a 1.77 gigapixel single image, achieved by scanning and stitching a series of close-up analog images captured from multiple camera viewpoints. The *Gigapxl Project* (2001) took a different approach. Led by Graham Flint, it used a camera with a large format film of ~23 by 46 centimeters, enabling the capture of four gigapixels in a single shot from one camera viewpoint without the need for stitching (Graham, 2006).

In the 2000's, the field of born digital gigapixel imaging was pioneered by Greg Downing and xRez Studio's ground-breaking *The Yosemite Extreme Panoramic Imaging Project* (2008). The team designed an automated motion control head to capture a large number of overlapping images from a single camera viewpoint. They also utilized telephoto lenses with higher pixel-per-degree density than standard and wide-angle lenses to acquire images of Yosemite Valley's granite walls with unprecedented detail. The project was remarkable for two primary reasons: it elevated panoramic gigapixel imaging from an artistic practice to a scientific endeavor by collaborating with geologists to document high-resolution baseline imagery for identifying and studying rockfall events in the valley (Xrez corporate author, 2010). The high-resolution dataset was further combined with terrain data acquired through remote sensing, creating a 3D model useful for scientific research and film production (Downing, 2010).

Bioscience and remote sensing have also adopted gigapixel imaging. BigTIFF (2004), the key file format for standardizing the storage and access of gigapixel images, is a commonly used by the bioscience community for example (Eichhorn, 2007). In microscopy whole-slide imaging routinely generates images up to several gigapixels, providing a glimpse into the mystical subcellular world beyond our natural vision. The Guinness world record holder for the largest digital image is a 281 gigapixel sagittal section of a 1.5 mm zebrafish embryo composed from electron microscopy images made in 2014, revealing intricate details up to the cell nucleus (Guinness World Records corporate author, 2010). Remote sensing also benefits from gigapixel imaging, enabling distant observation of the world with extraordinary details. Google Maps is exemplary in this field, being comprised of an 800 gigapixel stitched satellite image made in 2013 (Google, 2013). Another exciting possibility for capturing vast and detailed landscapes is drone aerial photography (a recent addition to remote sensing), demonstrated in Jamen Percy's 700 image stitched drone photography of the Black Rock City Arts Festival in 2022 (Growcoot, 2022). Remote sensing has additionally contributed to the development of GeoTIFF (1997), a specialized format for combining gigapixel data with geocoding information (Ritter and Ruth, 1997).

**Figure 2:**
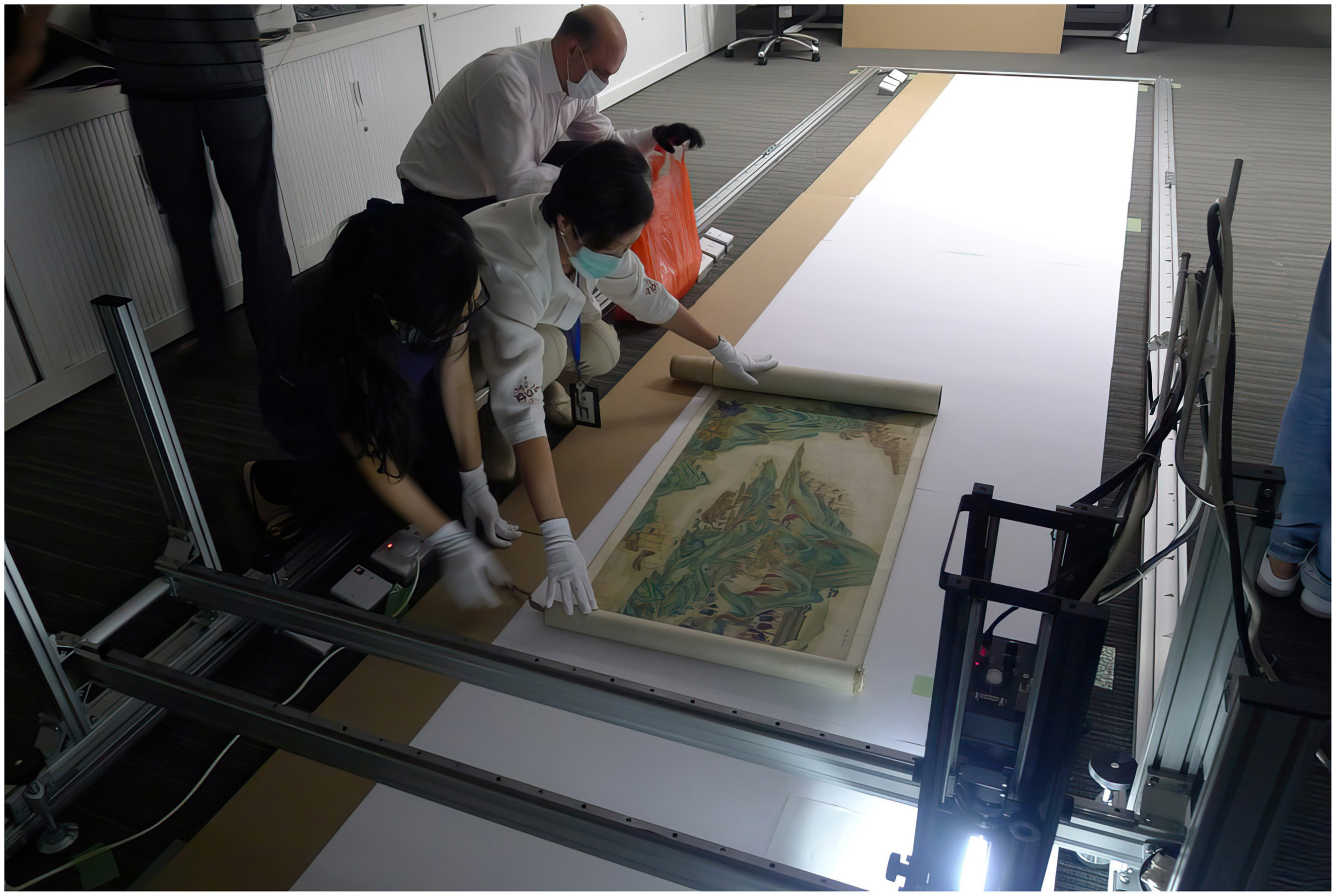
Digitizing the scroll painting Pacifying the South China Sea Pirates, Hong Kong Maritime Museum. Photo: Sarah Kenderdine.

**Figure 3:**
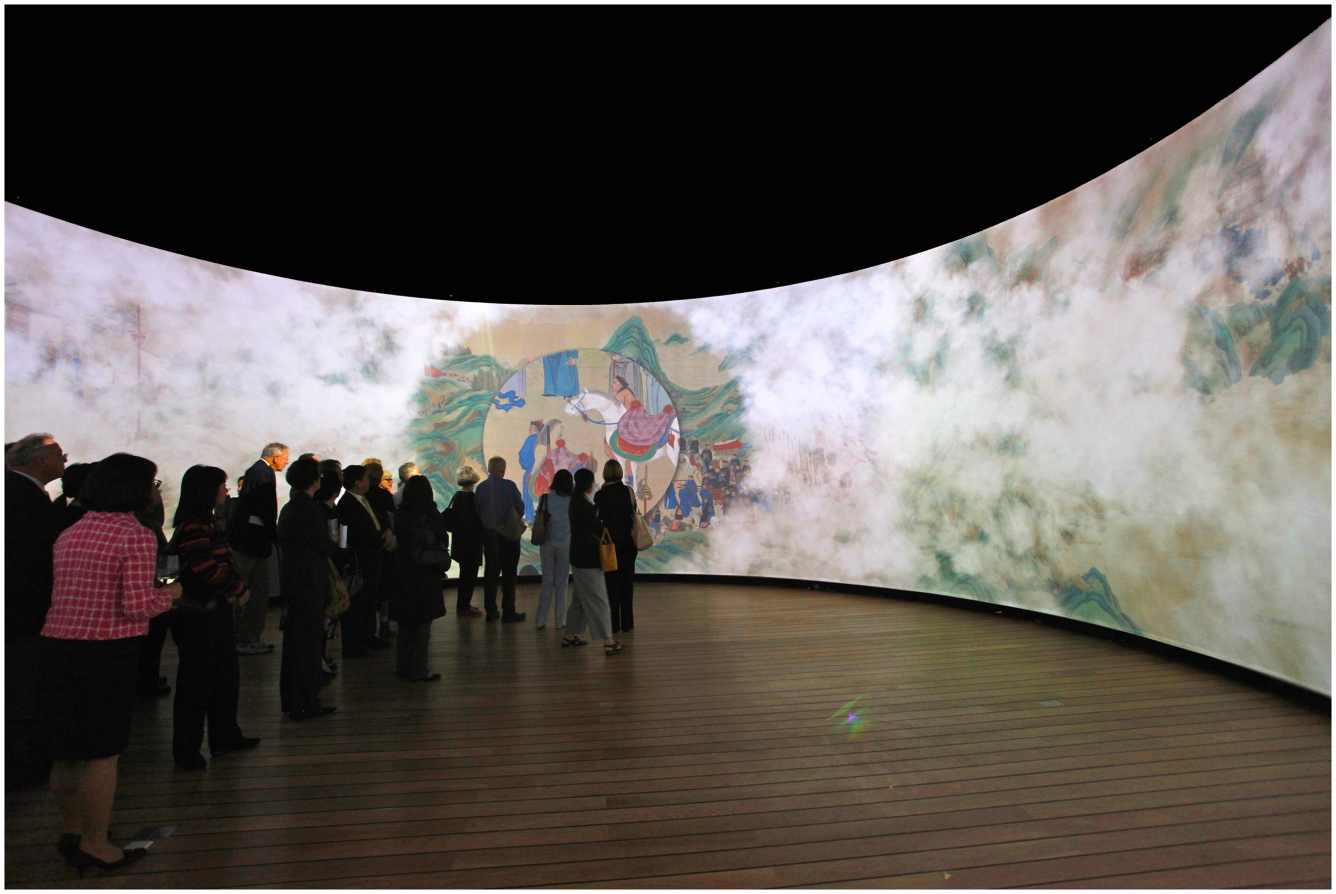
*We are like Vapors*, 360-degree interactive display of the Pacifying the South China Sea Pirates, Hong Kong Maritime Museum. Photo: Sarah Kenderdine.

Cultural heritage institutions such as museums, have also begun to use gigapixel images for the documentation, preservation, analysis, and dissemination of collection items. Various techniques, including single/multi-camera and single/multi-viewpoints setups, have been developed to digitize masterpieces in different contexts. Gigapixel images are predominantly utilized for digitizing flat objects, where a typical setup involves a single camera system on a movable, motorized mount attached to an imaging frame. In 2014, LUXLAB of the City University of Hong Kong and Kyoto University developed a technology capable of scanning flat up to 1,200 dpi using non-damaging light sources and a leveled imaging frame. This approach is particularly suitable for digitizing very long objects like hand scrolls, which are common in East Asian art but are very difficult to install or display (Figure 2). One example of this initiative is the digitization of the 18-meter-long hand scroll *Pacifying the South China Sea* (1810) in the collection of the Hong Kong Maritime Museum since 2004.^8^ The scroll is one of Hong Kong's most important tangible cultural heritage artifacts, depicting the Imperial navy's campaign in Guangdong, China, and the capitulation of Cheung Po Tsai (1783–1822), a legendary naval officer and former pirate in Hong Kong. The completed gigapixel image was the basis for two subsequent interactive installations. The first version, *We are like vapors* (2013), presented the scroll as a 360-degree immersive projection (Figure 3) with an augmented animation revealing the twenty scenes of the scroll (Kenderdine, 2013). The second version, *The Scroll Navigator* (2013) features a downsized photograph of the scroll with a motorized LCD monitor mounted on a track above it. This system allows visitors to control their movement through the scroll using a handheld iPad, while simultaneously illuminating the corresponding section on the scroll (Kenderdine, 2013). The combination of gigapixel imaging and interactive visualization serves in this instance as a powerful tool to unveil details and narratives that would usually be unattainable using conventional museum apparatus.

In 2022, the Rijksmuseum in Amsterdam released the 717 gigapixel (925,000 by 775,000) digital image of Rembrandt's (1606–1669) masterpiece *The Night Watch* (1642), measuring 3.78 by 4.53 m. Generated from 8,439 individual shots, this gigapixel digital representation currently stands as the largest single image ever created for an artwork (Rijksmuseum corporate author, 2019). The 5.6 terabyte image was captured using a 100 megapixel Hasselblad H6D 400 MS camera, mounted on a dynamic imaging frame with vertical orientation. Accompanied by data from macro X-ray fluorescence scanning (macro-XRF), hyperspectral imaging, and infrared reflectance imaging spectroscopy (RIS), this gigapixel image has allowed the museum to investigate the painting's condition and make plans for its conservation, undertaken 2 weeks after the release of the gigapixel image. Furthermore, the high level of detail has facilitated studies into how the masterpiece was painted, including insights into pigments and chemical composition (Rijksmuseum corporate author, 2022).

There are however significant challenges to producing a gigapixel image of a painting that is vertically oriented, and which may not be entirely stationary (Gabrieli et al., 2021). Perhaps the most innovative achievement with this gigapixel image is the restoration of the missing part of *The Night Watch* accomplished using artificial intelligence by the scientists from the Rijksmuseum. Around 73 years after its completion in 1715, the painting was moved to the town hall of Amsterdam, which unfortunately resulted in cropping the work to accommodate its new location. The detached sections of the artwork were then lost. This would have made the original appearance of the painting a mystery, except that the artist Gerrit Lundens (1622–1686) had made a miniature copy of *The Night Watch*, which survives to this day. Both the original work and Lundens' copy have provided data for scientists from the Rijksmuseum to train neural networks to predict how Rembrandt would have been painted the missing parts of *The Night Watch*. The restored parts were subsequently, printed and mounted to recreate the painting. The gigapixel image played a crucial role in the success of this restoration, as unparalleled level of detail of finely preserved brushstrokes resulted in the remarkable quality of the machine's replication of Rembrandt's gestures.

## 4 Omni-directional visualization of cultural big data: the digital twin of the panorama of the battle of Murten (1894)

**Figure 4:**
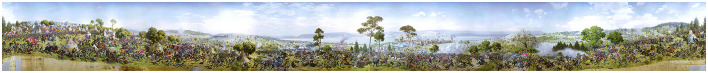
Louis Braun, Murtenschlacht Panorama, 1893–4. Oil painting on canvas, 10 x 100 meters. Image credits: EPFL, eM+, 2023.

*Digitizing and Augmenting the Panorama of the Battle of Murten* project is led by the EPFL Laboratory of Experimental Museology with the Foundation for the Panorama of the Battle of Murten. The original Murten panorama (Figure 4) is a Swiss national treasure (Schaible, 1997). It was realized by the renowned German panorama painter Louis Braun (1836–1916) between 1893 and 1894, commissioned by the Swiss company Schweizerische Panoramagesellschaft.^9^ During the year-long production of this massive work, which measures 10 by 100 m, around 40 painters were active in Braun's workshop. The painting depicts the moment that Swiss Confederates and their allies gained the upper hand against the army of the Duke of Burgundy in 1476, during a battle that changed European history and defined Swiss identity. Originally displayed periodically between 1894 and 1909, in rotunda theaters in Zurich and Geneva (Fritz and Schwarz, 1997, p. 40–41), it later vanished from Swiss public consciousness. The panorama was only restored in 2002 (Schaible, 2002) to be placed on display for 4 months at the 2002 Swiss National Exhibition (Expo.02), after which it was relegated to permanent storage.

The DIAGRAM project has two aims: the first is to digitize the original artwork, the only nineteenth-century Swiss panorama that remains inaccessible to the public; the second is to display the digital twin in large scale immersive and interactive digital visualization systems and augment it with new features for access and interpretation. The digital exhibition of the battle of Murten panorama will also benefit from sophisticated interpretation approaches to contend with a range of contested histories. These include the commemoration of a battle that comprises a constitutive element of Swiss identity that was foundational to political discourses at the end of the nineteenth century and during twentieth-century world wars, while the social significance and cultural appropriation of this image has critically changed in the twenty-first century. The second element requiring deconstruction is the nineteenth-century visual representation of a medieval battle, which has been clouded by contemporary fascination with the Middle Ages (medievalism). The project sponsors, the research team and the museum partners intend to open this work up to researchers and the public to critically interpret the panorama's digital twin. In this regard, the digital twin and its augmentations will offer new approaches to cultural big data visualization, based on the original form of the painted panorama, the amount of data linked and generated, as well as through the immersive experience for viewers.

### 4.1 The largest digital twin of a single object

The Murten panorama's digitization process centers on an ultra-high-resolution camera (Phase One, iXH 150-megapixel camera with a 72-millimeter MKII lens). The imaging target was 1,000 dpi (40 pixels/mm) and required 27,000 photographs. Each photograph captured a region of 360 by 270 millimeters and incorporated sufficient overlap with its neighbors for the subsequent stitching process. Industry standard software designed for processing 360 panoramas, known as PtGui, was used to align and detect feature points and stitch the individual photographs together. These methods have produced the largest single seamless digital image of a painting ever created, at 1,600 gigapixels. In terms of storage space, it occupies 9.6 terabytes as a 16-bit RGB uncompressed BigTIFF file (1,600,000,000,000 by 3 by 16 bits).

Accessing this colossal image poses significant challenges, primarily because the size of the file exceeds the capacity of a computer's memory. To address this issue, image pyramid and tiling techniques have been employed to create a multi-level representation of the panorama. The process entails each level in the pyramid being scaled down by a factor of four (a factor of two for each dimension) compared to the previous level, a process that continues until a minimum image tile size is reached. This approach allows for seamless zooming and panning experiences by streaming only the required tiles from the optimal zoom level. Following the image pyramid process, the digitized panorama will comprise a maximum of 23 levels and 32,570,306 tiles (at 256 by 256 pixels per tile) and contain a further 3.2 terabytes of pixel data, bringing the total image size to 12.8 terabytes.

Measuring 717 gigapixels, the 2022 digital twin of *The Night Watch* constitutes <½ the number of pixels of the Murten Panorama, which will soon hold the distinction of being the largest single digital image of an artwork. Apart from the difference in scale of the original paintings, the resolution of the sensor and the imaging processes are two other notable distinctions between these projects.

**Figure 5:**
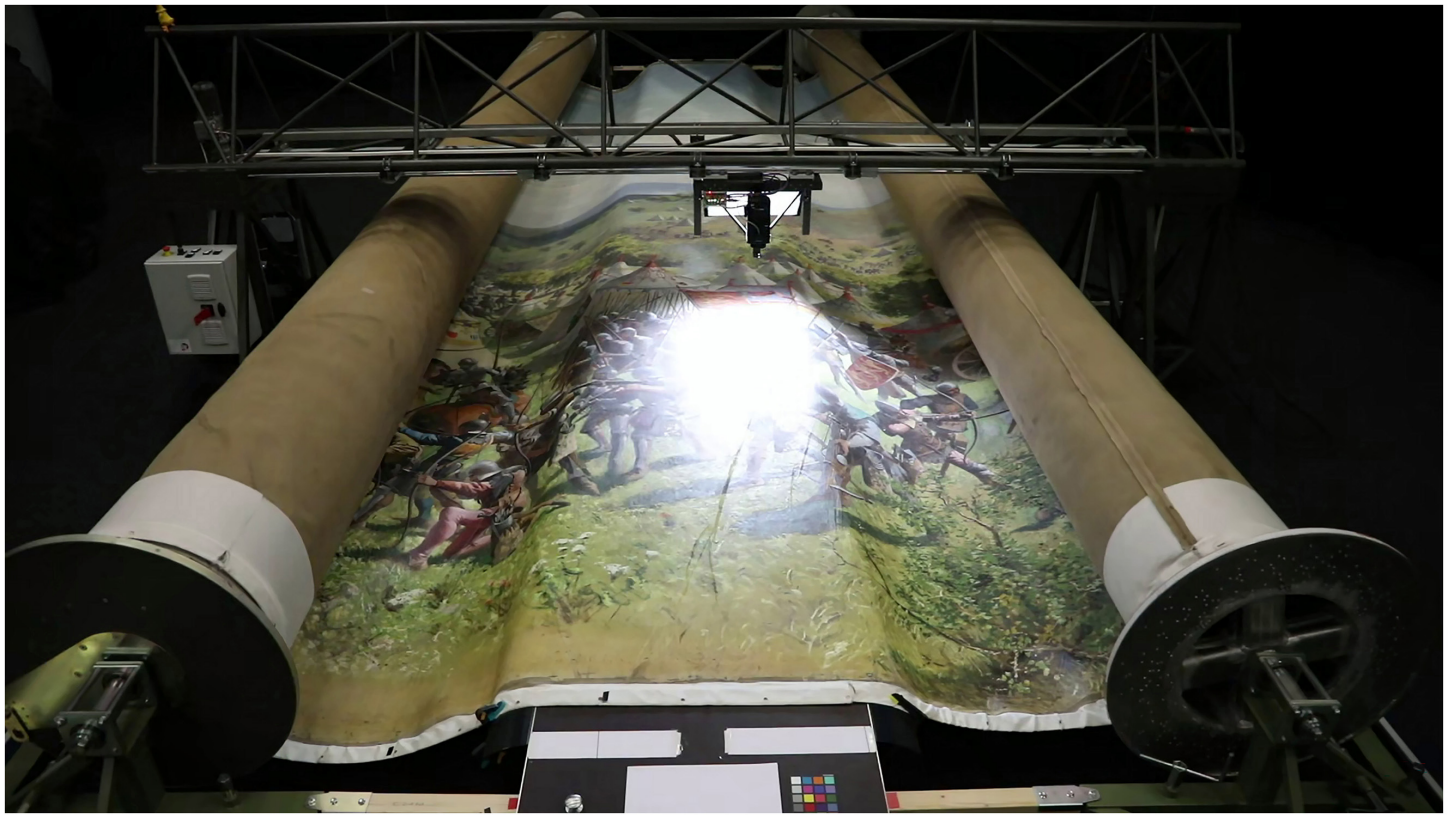
Setup for the imaging process of the Murten Panorama (2023). Image credits: EPFL, Laboratory for Experimental Museology.

The imaging configuration to capture the digital twin of the Murten panorama (Figure 5) consisted of a horizontal platform to support a section of the canvas, which measured 1 by 11 meters. An elevated motorized rig provided computer-controlled positioning in two dimensions above the painting with accurate and repeatable spatial positioning (±0.01 mm). The motorized rig hosted the Phase-One iXH (150-megapixel) camera with a 72 mm MKII lens, an Akurat D8 MK2 lighting system and a motion/vibration sensor. Both the camera and the raking light (angled at 45°) were therefore on a constant plane and at a fixed vertical distance from the painting with the optical axis of the camera perpendicular to the painted surface. This setup corresponds to a method known as the parallel-multi-viewpoint capture technique (Cabezos-Bernal et al., 2021).

Lens cast calibration (LCC) was also employed to compensate for illumination variation across each photographed area, which was achieved by photographing a white piece of paper at the start of each section. Color calibration was additionally performed at the start of each section with a Spyder color chart. Cross polarization, light and a lens linear polarizing filter at 90° to each other, were used to remove specular highlights arising from the paint and the occasional metallic insets.

The Murten panorama was originally divided into three rolls, which underwent conservation prior to the imaging.^10^ During the imaging, each roll was placed adjacent to the imaging platform and a one-meter-wide section laid across the platform. For each section that was digitized, the computer-controlled rig positioned the camera in a regular grid of four columns by 58 rows. Once the vibration control indicated the camera was stationary at each position, the photograph was taken and immediately transferred to computer storage. At the conclusion of this automated procedure, the 232 photographs were checked by stitching them together along with the previous one-meter section. Once proven successful, the canvas was manually advanced by 1 m.

### 4.2 Augmenting the panorama: the dataset and its visualization strategies

The Murten panorama features a rich array of cultural elements, including geolocations, heraldic representations, historical characters, events, armaments, and costumes. There are two main discourses on the interpretation of the panorama: the historical discourse, focusing on the deconstruction of the contested history depicted in the painting, and the artistic, explorable, and enjoyable nature of the masterpiece.

A scholarly platform will be developed for the painting's historical discourse, which will support the annotation of the panorama using Linked Open Data at a local level. On this platform, textual and visual contextual materials will be represented as information objects using CIDOC-CRM and its extensions. These materials will then be associated with fine-grained semantics related to points of interest observed in the panorama, utilizing precise image region URIs and SVG masks defined by IIIF Image API (Snydman et al., 2015) and the Web Annotation Data Model (2017) standards. This process will create an interoperable, linkable, and computable interpretation layer on top of the gigapixel image of the Murten panorama. This annotation model is also applicable to other genres of motif and narrative rich intricate visual culture, such as cabinets of curiosities or Kunstkammers and mural paintings. The resulting web of art historical information will be even more fine-grained compared to current iconographical databases, such as Arkyves (see Brandhorst and Posthumus, 2014) and the Warburg Institute Iconographic Database (Warburg Institute, 2010). Annotation up to the local level will further enable new research avenues, such as the weighted network analysis of different motifs and patterns over time.

The second discourse relates to the cultural big data visualization strategies. The key question is how to empower audiences to browse the panorama based on their personalized paths, given its incredibly rich content. When the panorama is unpacked into a database of entities (person, weapon, and animals), it resembles the same scenario of searching a substantial quantity of datapoints. Although it can be argued that panning across the vivid panorama itself is already an enjoyable experience, this could be extended by offering a generous browsing interface based on clustering visual features such as postures, costumes, and emotions. Overlaying these cluster maps on the panorama could reveal the hidden dynamics embedded within it, while allowing the audience to explore the image in depth using the map as another affordance. Notably, this type of visualization can also support research activities, as demonstrated by *Blue Dots 360*. Another approach involves the datafication of the panorama, providing users with a chatbot guide grounded in the vision-language embedding of the panorama. This approach is similar to the natural language interface for video retrieval proposed in the project to enhance access to the RTS video archive (see Yang, 2023a,b; Yang and Zhang, 2023).

Both these approaches present significant challenges in computer vision due to the immense image size, high entity density, and variation in scale. The initial task in the processing pipeline, before object detection or applying vision-language embedding, is thus to extract optimal contextual windows from the panorama. Despite considerable research and the promising performance of high-resolution deep learning in fields such as medical imaging, remote sensing, and surveillance (Bakhtiarnia et al., 2024), there are still some obstacles to overcome. For one there is a lack of training data at a scale and semantic composition comparable to large and intricate paintings, as evident in the current state-of-the-art dataset for human-centric scenes, PANDA-Crowd dataset at 26,558 by 14,828 pixels (Wang et al., 2020). This problem combines with the domain shift issue for applying pre-trained vision foundation models on painting, indicating that there are still gaps to be bridged for high-resolution deep learning in the context of cultural big data.

**Figure 6:**
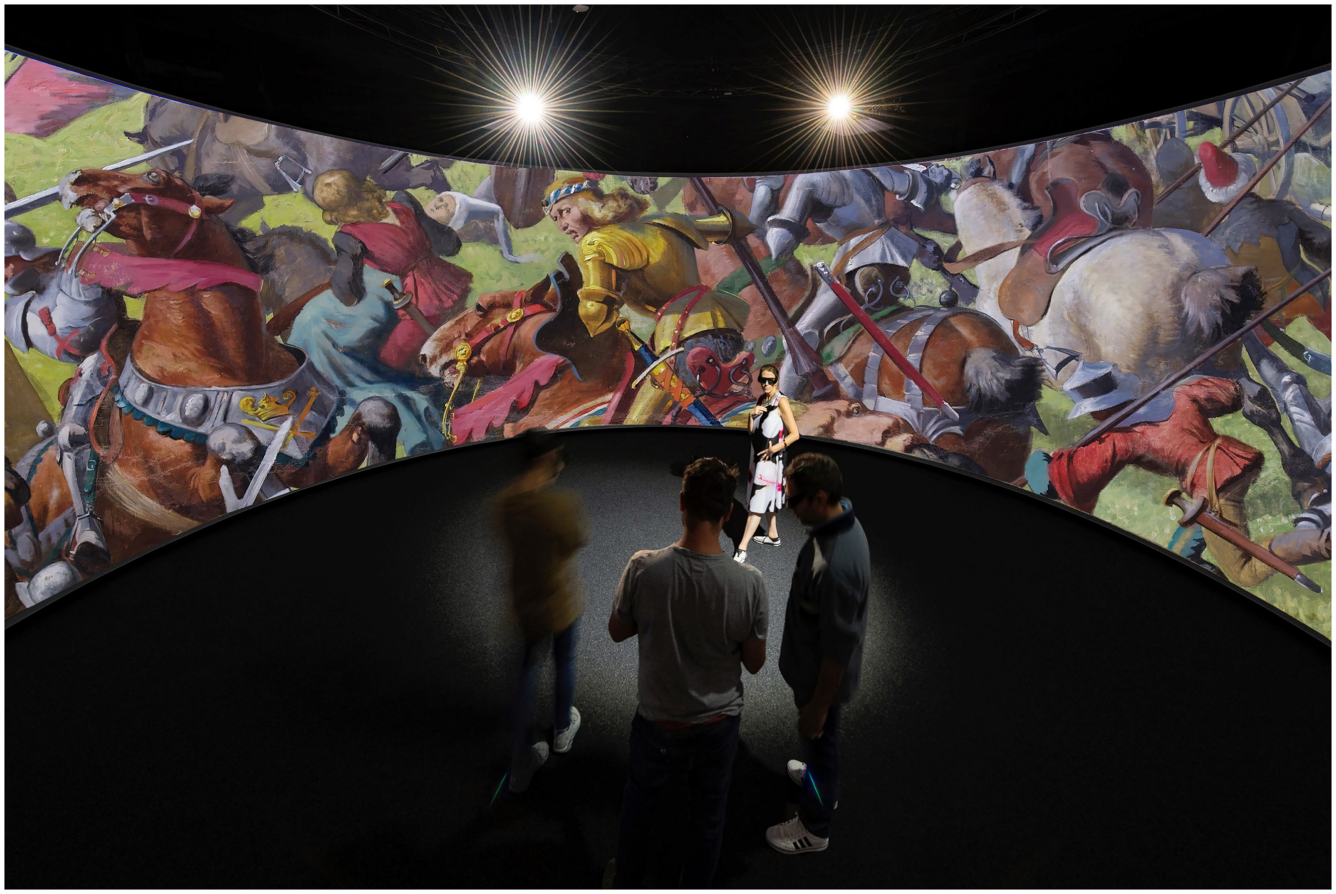
Panorama+, large scale visualization system 360-degree 3D, photographic montage of the digital twin of the Murten panorama in the Panorama+. Image credits: EPFL, Laboratory for Experimental Museology.

Experiencing the Murten panorama's ultra-high-definition digital twin in a large-scale 360-degree 3D projection system known as Panorama+ (Figure 6) with a three-degree of freedom interface (3DOF) will allow viewers to freely pan across or zoom into the image.^11^ The Panorama+ system allows for up to 20 participants in its ample space of 10 m diameter by 4.8 m high. The detail revealed in the gigapixel image transcends the human view of the original panorama, conventionally staged as part of an observation platform that also kept viewers far from the canvas. The digital twin, in contrast, has 20 levels of image zoom that are synced with an ambisonic dynamic soundscape, maximizing the immersion effect and narrative flow for viewers navigating the painting.^12^ Synthetic smells will be emitted on collars worn by visitors, synced with the portion of the panorama on view. These innovations will be tested in the EPFL Laboratory for Experimental Museology in 2024 and presented to the public in partner museums in 2025–2026.

The DIAGRAM project has already made many achievements in terms of visualizing cultural big data, but the work has further implications. As previously mentioned, the contested nature of its interpretation makes the contemporary standalone presentation of the image in a museum context problematic. Different features are currently being produced that will accompany the image to permit different perspectives on the past. This includes different types of data that could be used to augment the image as part of the Panorama+ application, or to conduct analysis via a text-based user interface (TUI) or a graphical user interface (GUI), intended for scholarly audiences, or a in a curated version for public presentation. The premise is to offer the means to visualize data or to consult by reading, outside the virtual experience to allow a certain critical distance for the interpretation of the image.

**Figure 7:**
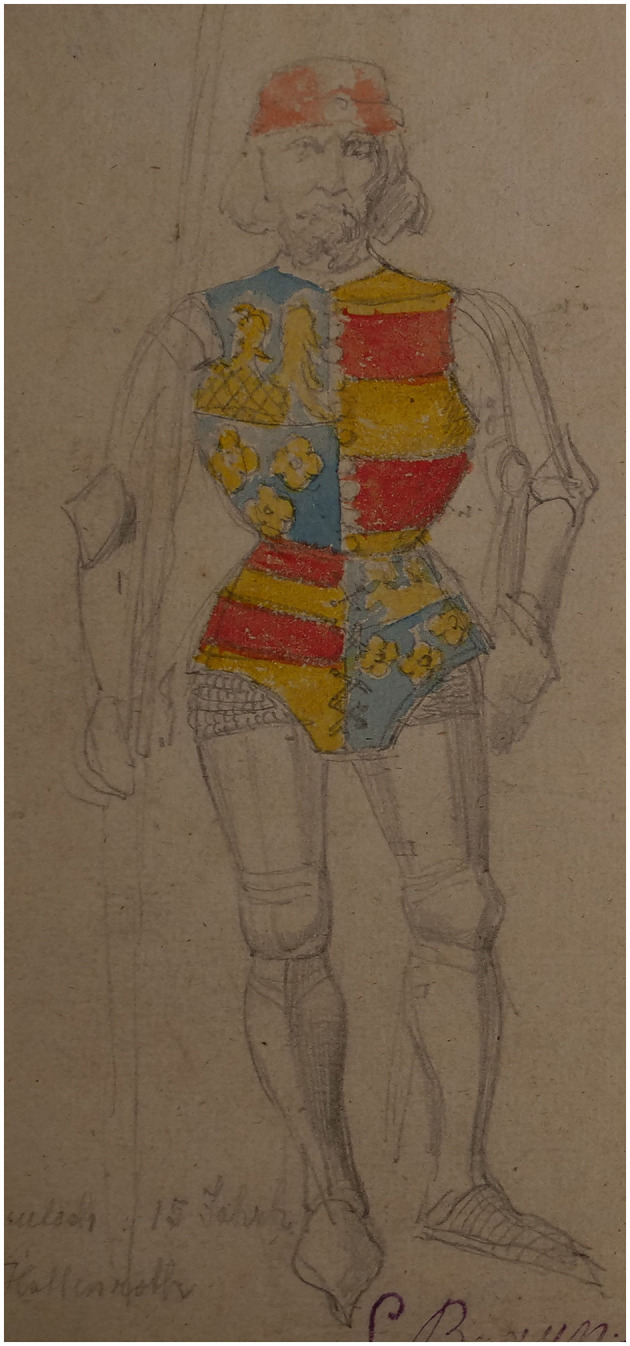
Louis Braun, preparatory drawing, 1893 (Schwäbish Hall, Hällisch-Fränkisches-Museum). Center panel. Photo: Daniel Jaquet.

**Figure 8:**
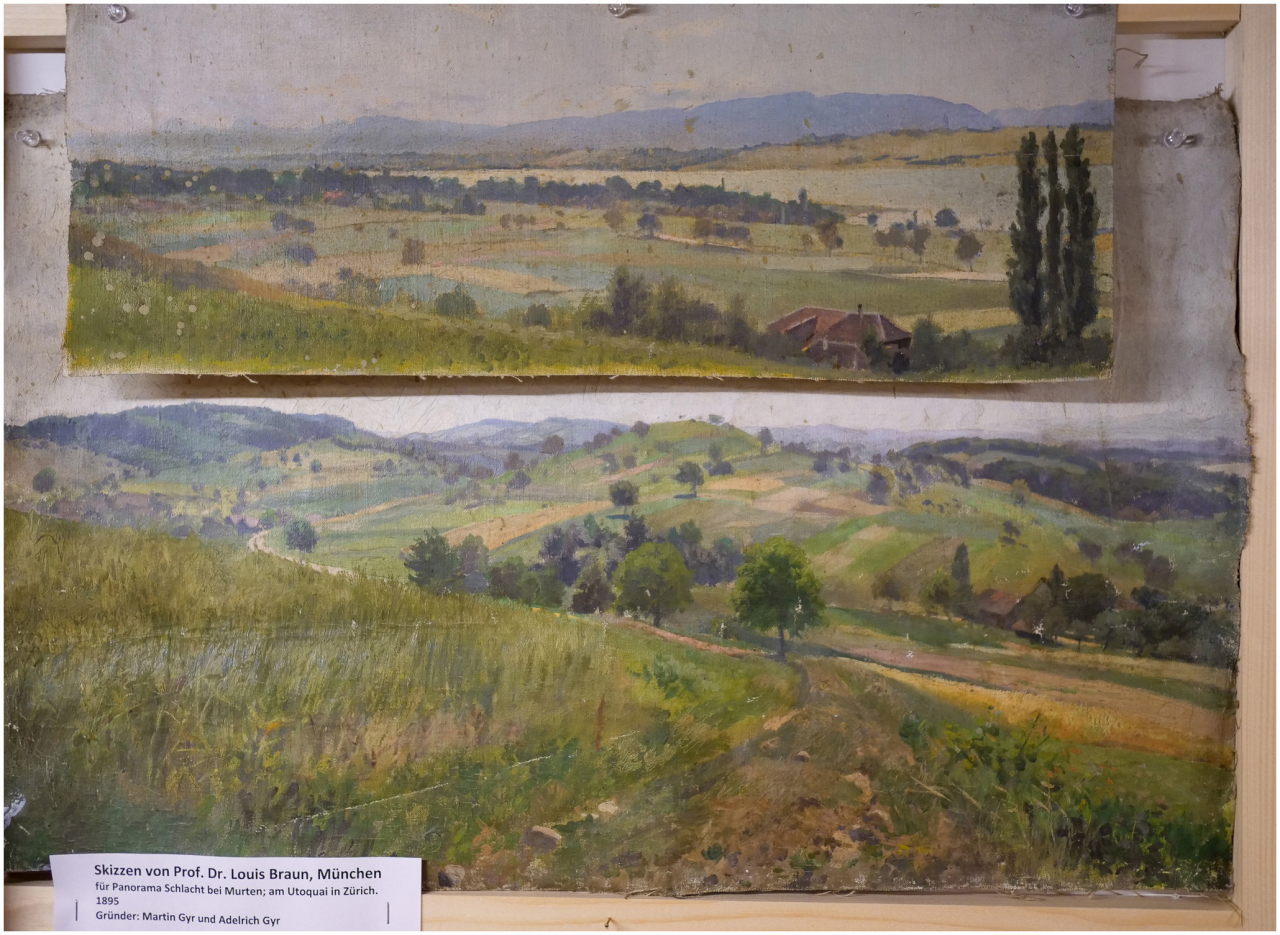
Louis Braun, landscape study of Murten (1893; Switzerland, private collection). Photo: Daniel Jaquet.

**Figure 9:**
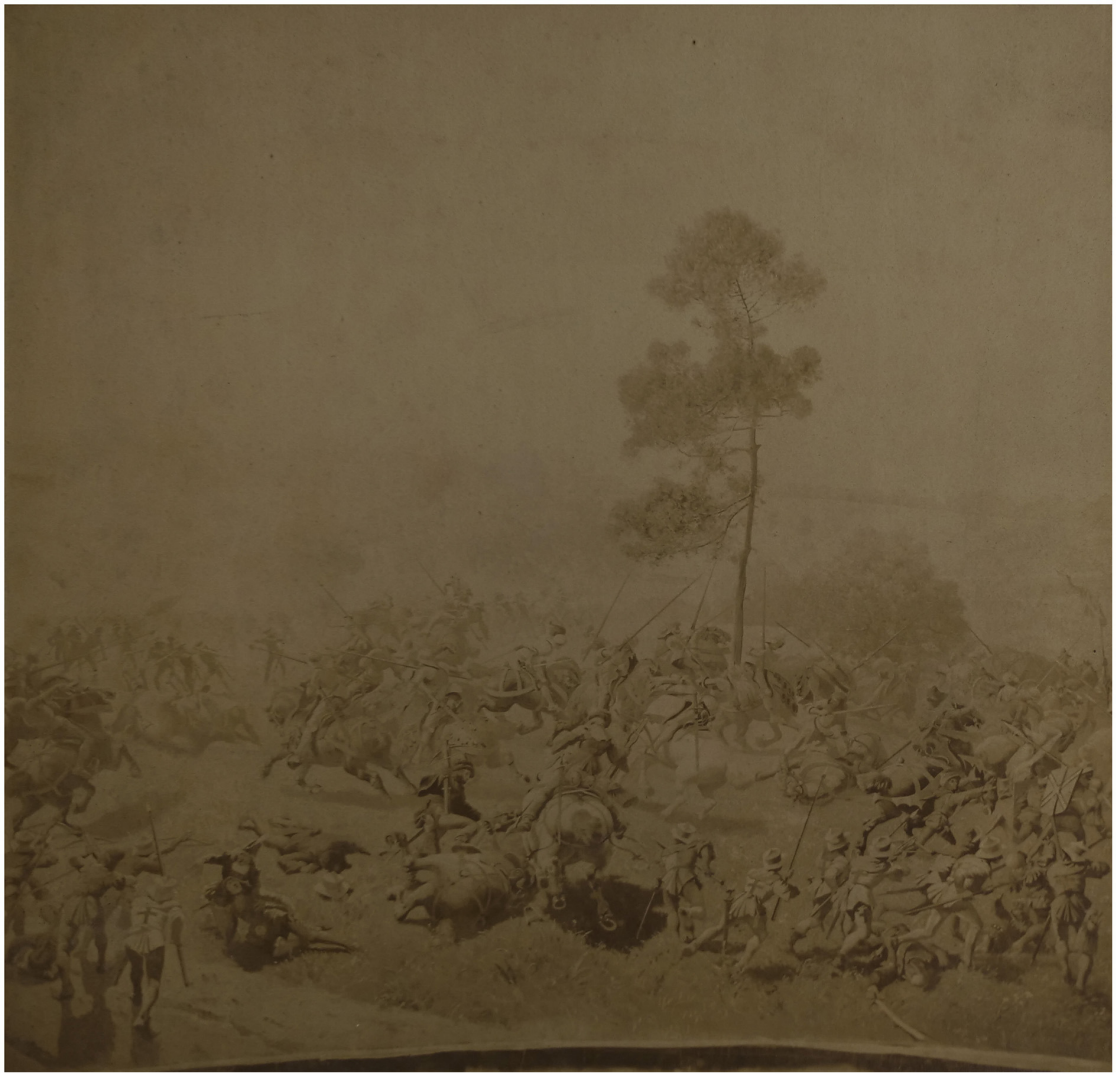
Early photograph of the Murten Panorama, non-dated (early twentieth century; Schwäbish Hall, Hällisch-Fränkisches-Museum). Photo: Daniel Jaquet.

An added overlay format consists of superimposing topographical model and images of the contemporary landscapes of Murten, viewed from the same perspective as in the painting. Examples of additional data include 3D models of archival objects, such as arms, armor, accessories, to allow for comparison with those illustrated in the painting. Reconstructed 3D models of costumes, based on studies of late Medieval costume patterns will enable the close observation of the 2D representation of costumes from different angles.^13^ Volumetric 3D motion capture of actors re-enacting gestures from selected scenes in the painting will be created, which will also be vital to overcome the lack of parallax when observing a still image representing movement. All the 3D animations will be overlaid on the original image in the application for the Panorama+ installation, which will be re-scalable for augmented or virtual reality experiences. Documents will be digitally linked to points of interest in the painting. This will include archives of iconographical documents related to the production and exploitation of the panorama, as well as the late-Medieval battle itself, such as paintings, illuminations, preparatory drawings, and early photographs (see Figures 7–9) alongside text-based documentation, including chronicles, letters, and historical accounts.^14^

The overlay will also further permit the limitless addition of text-based metadata, documenting the augmentation work and providing access to relevant data. Digital images and datasets are inherently malleable and can be adapted to different viewing contexts, using different visualization systems. In the instance of the DIAGRAM project, the installations, systems and content are being curated by museum experts in collaboration with the research team, offering the means for a critical interpretation of the work to their targeted audience. For example, the differences between a painted representation of an object rendered in the nineteenth century can be compared with an ultra-high fidelity 3D model of an original fifteenth-century object held in a public collection, an ultra-high-resolution scan of an iconographic representation from the fifteenth century, or an edition of textual descriptions from the fifteenth century. Each 3D model, image or text can be accompanied by metadata providing essential descriptive information or relaying the scientific research and historical discourse associated with its study. Another example of critical interpretation is the addition of information about the context in which the image was produced or viewed in the nineteenth century. These additions may be textual, visual or sonified. They can also be included in the virtual experience or take the form of analog or digital installations situated adjacent to the virtual experience in the museum space. A custom-built website is finally envisaged for even wider dissemination and for re-use of this work for other contexts, such as archiving, communication or research into the digital object.

## 5 Conclusion

The original research presented in this article has focused on the DIAGRAM project's digitization, visualization and augmentation of the Murten panorama, as highly significant yet contested Swiss cultural heritage treasure. The various objectives of the project have been analyzed to explore what is possible to achieve with a gigapixel cultural heritage image, particularly in relation to the historic and cultural aspects of the original object. The size of the painted panorama, itself an enormous canvas, in conjunction with the highest available resolution in the experimental imaging process, has given rise to a very large image and big data. The focus on producing high-end museum experiences in large scale 360-degree 3D projection systems as an output of the project further prompted the research team to conduct research in the field of mass media phenomenon in the nineteenth and twentieth centuries, as described in this article. This naturally led the research team to use a panoramic visualization system to prioritize the inclusion of essential elements that enhance immersion, such as sound and smell, in addition to all the other features of the viewing experience and the augmentations.

The initial survey of the history of the panoramic format identified the strategies of visualization as a distinct cultural phenomenon of the nineteenth century. The size of the image, its interaction design, as well as the documents at the disposal of the user in the experiences of the nineteenth and twentieth centuries were however limited by the technological and financial resources of the time. With digital technologies, many of these limits have been overcome to allow access to interconnected data within or alongside the visualization experience. It is nonetheless important to remember that panoramic visualization is not a question of making everything available for all data visualization experiences. The choices regarding the visualization tools, the interaction design, and the type and amount of data accessible need to be tailored to each specific cultural collection or object and its audience, location, and purpose. The Laboratory for Experimental Museology has developed multiple strategies for visualizing large datasets for immersive, omnidirectional experiences in large-scale museum installations. These have been extended for the DIAGRAM project, for which we created the dataset, considered cultural big data, and visualization strategies for the digital twin of the Murten panorama.

This article has further examined the transition to digital technologies to explore the new affordances offered in the use of gigapixel images and the panoramic format in the cultural heritage sector. The possibilities that gigapixel images offer fields of conservation and restoration have also been amply studied, as delineated in this article. Some, if not all, gigapixel imaging projects in the past 20 years have pursued the technical challenge of producing and viewing very large-scale and ultra-high-resolution images. These parameters are not however the primary objective of the original research undertaken for the Murten panorama, which is focused on innovation in the field of cultural heritage dissemination through advanced or experimental visualizing systems. Rather, the DIAGRAM Murten panorama project has inspired new areas of research into the affordances of gigapixel image of a cultural object developed for a panoramic cultural heritage visualization system. As the project prioritizes the materiality of the original object, including the augmentation of the image and its support, it has generated unprecedented approaches for interacting with and discovering the rich features of the panorama. Most notably, audiences will be able to explore the Murten panorama's complex and massive image through interpretive layers and augmented strategies that enhance immersion in the viewing experience as well as critical interpretation of its content. These novel approaches are additionally paving new modes of sustainability for the panoramic visualization of gigapixel images in digital panoramas as well as the emergence of new ones.

## Data Availability

The raw data supporting the conclusions of this article will be made available by the authors, without undue reservation.

## Footnotes

^1^Swiss exhibition locations include the Castle of Grandson, Murten Museum, Museum für Gestaltung, and Bernisches Historisches Museum (BHM). Other locations are planned, see the webpage of the project DIAGRAM, eM+, EPFL 2022-6. Available at: https://www.epfl.ch/labs/emplus/projects/diagram/ (accessed June 21, 2024).

^2^The patent specified not only the vast circular painting, but also the building in which it was to be exhibited.

^3^For a recent deconstruction of the features of the illusion in panorama installations (painting, photography, or digital), see Asisi (2019). About the contemporary reception of the panorama, see Huhtamo (2013), Oettermann (1997), and Sternberger and Neugroschel (1977).

^4^Two of the largest preserved panoramas are the Raclawice Panorama (1894) kept in Wroclaw, and the Mesdag Panorama (1880) kept in The Hague, both measuring 15 x 115 m. There were larger panoramas documented, especially moving panoramas, up to 6,437 m long (Richard Risley, Professor Risley's Gigantic Moving Panorama of the Mississippi River, 1853). Among the smallest, a good example is the Panorama of Rome by Ludovico Caracciolo, painted in 1824 (London, Victoria and Albert Museum).

^5^The Cosmorama was installed in the Palais Royal from 1808 to 1828 and moved to the Rue Vivienne from 1828 to 1832. After 15 years of operation, this Cosmorama showed over 800 paintings of about 113 by 81 cm, displayed across 80 m, changing every month (Huhtamo, 2019).

^6^Both the leaflet and the original manuscript were consulted in the archive collection of Louis Braun, at the Kulturhistorische Hällisch-Fränkische Museum, Schwabish Hall, Germany (shelf number 2015_1001, 104, and 105). The final text is short (1,760 words).

^7^A square image of 31,623 x 31,623 pixels represents 1 gigapixel.

^8^Pacifying of South China Sea, 1810. Hong Kong Maritime Museum, HKMM2004.0102.0001.

^9^Later displayed in Geneva in 1904, and then in Zurich in 1907 for its last presentation.

^10^A 6-year conservation and restoration project (1996–2001) was completed before being exhibited to the public for four months during the national exhibition in 2002 (Expo.02), see Schaible (1997, p. 24–27) and the conservation report (Archive of the Foundation for the Panorama of the Battle of Murten, held in the National Library Bern, Switzerland).

^11^Panorama+ is a system developed by Sarah Kenderdine at the Laboratory for Experimental Museology. The panorama screen is based on UNSW iCinema Research Centre's landmark 360-degree stereoscopic interactive visualization environment—AVIE (Advanced Visualization and Interaction Environment). The standard configuration is a cylindrical projection screen four meters high and ten meters in diameter, with a twelve-channel stereoscopic projection system and a 12.2 surround sound audio system. AVIE's immersive mixed reality capability articulates an embodied interactive relationship between the viewers and the projected information spaces. The EPFL panorama+ has been re-designed to increase the resolution four times to an effective resolution of 22,000 pixels by 1,750 pixels in 3D.

^12^The production of the soundscape is a separate project in partnership with the DFG project *Der laute Krieg und die Laute des Krieges. Belliphonie im Mittelalter* (2021–2024), led by Prof. Martin Clauss (Technische Universität Dresden).

^13^This part of the project is being led by Prof. Kathles en Martin of Drexel University.

^14^The basis for this work is the edition of sources related to the battle (Ochsenbein, 1876), in addition to ongoing research by the project team.
